# The effect of self-care education program on the severity of menopause symptoms and marital satisfaction in postmenopausal women: a randomized controlled clinical trial

**DOI:** 10.1186/s12905-022-01653-w

**Published:** 2022-03-14

**Authors:** Leila Karimi, Maliheh Mokhtari Seghaleh, Robabeh Khalili, Amir Vahedian-Azimi

**Affiliations:** 1 Behavioral Sciences Research Center, Life Style Institute, Nursing Faculty, Baqiyatallah University of Medical Sciences, Tehran, Iran; 2 Trauma Research Center, Nursing Faculty, Baqiyatallah University of Medical Sciences, Tehran, Iran

**Keywords:** Education, Self-care, Quality of life, Postmenopause, Clinical trial

## Abstract

**Background:**

Physiological and psychological changes during menopause can affect the quality of marital satisfaction. The aim of this study was to evaluate the effect of self-care education program on the severity of menopause symptoms and marital satisfaction in postmenopausal women.

**Methods:**

In this randomized controlled clinical trial, 70 postmenopausal women who referred to the gynecology clinic of Baqiyatallah and 502 Artesh hospitals in Tehran, Iran, and met the all inclusion criteria were randomly allocated into two equal groups (intervention and control groups) using block randomization. The intervention group received self-care training program in physical, psychological, social and sexual dimensions in 5 sessions during a week. The control group also had 5 sessions exactly the same as the intervention group, except that they received only routine care and training. Data were collected pre- and post-intervention using Menopause Symptoms' Severity Inventory (MSSI-38) questionnaire and the Revised Dyadic Adjustment Scale (RDAS) questionnaire.

**Results:**

In the control and intervention groups before the intervention, socio-demographic characteristics (*P* > 0.05), the mean scores of MSSI-38 (*P* = 0.388) and RADS (*P* = 0.476) were not statistically significant. However, in the intervention group the mean scores of MSSI-38 (49.88 ± 3.3 vs. 39.33 ± 3.7, *P* < 0.001) and RADS (35.15 ± 4.3 vs. 49.48 ± 3.2, *P* < 0.001) after the intervention changed significantly and this change were statistically significant compared to the control group. Significant inverse correlation between severity of menopausal symptoms and marital satisfaction was observed with r = -0.461, *P* < 0.001.

**Conclusion:**

Our findings indicate that self-care training has a positive effect on the severity of menopause symptoms and also improves marital satisfaction in postmenopausal women. Therefore, we recommend that more attention be paid to providing self-care educational content to improve the marital satisfaction in postmenopausal women.

*Clinical trial registration* Iranian Registry of Clinical Trials; https://www.irct.ir/trial/49225 (IRCT20200624047910N1), registered (10/11/2020).

**Supplementary Information:**

The online version contains supplementary material available at 10.1186/s12905-022-01653-w.

## Introduction

Middle age is a period when most women experiences different changes. These changes can be social, physical, psychological, and economical. Moreover, in middle age, various chronic diseases such as hypertension, diabetes, arthritis and heart disease may appear and affect person [1]. This is also the time when women enter menopause and its symptoms including physical and psychological changes, affect their health and quality of women’s life [2]. Menopause is a normal stage in a woman's life that is recognized by the permanent cessation of menstruation after 12 months of amenorrhea [3, 4]. It occurs due to the exhaustion of the ovarian follicles, which leads to an increase in gonadotropins and a decrease in estradiol [5].

As a woman grows older, the number of ovarian follicles decreases. Decreased ovarian granulosa cells, the main producers of estradiol and inhibin, increase follicle-stimulating hormone (FSH) and luteinizing hormone (LH) production by not inhibiting the gonadotropin estrogen and inhibin. FSH levels are usually higher than LH levels because LH is removed faster than blood. Decreased estrogen levels destroy the hypothalamus-pituitary-ovarian axis. As a result, the endometrium stalls and the menstrual cycle become irregular until they stop completely [6]. This brings vasomotor and psychological symptoms [7]. Women may experience symptoms such as hot flashes [8], night sweats [9], mood swings, heart palpitations [10], bone pain [11], atrophy of the vagina and vulva [12, 13], and some other complications such as osteoporosis [14]. Experience depression, anxiety, stress and a decreased sense of well-being during this period, which will definitely affect the quality of life of these people [15]. Many factors play a role in bringing about those complications. One of these factors is age as a natural phenomenon driven by genetic and environmental causes. Menopause usually occurs between the ages of 45 and 55 and is known as the time of change from one stage to another in a woman's life [16].

One aspect of quality of life is the quality of marital relationships that may be disrupted during menopause [17, 18]. Marital satisfaction can be defined as an individual's attitude toward his or her marital relationship [19]. The quality of marital relationships has a multidimensional meaning and includes relationships between couples such as compatibility, happiness, life satisfaction, cohesion and commitment [20, 21]. The severity of menopause symptoms (e.g., hot flashes, night sweats, sleep disturbance, mood issues, vaginal dryness, sexual pain), may also impact a woman’s partner and her relationship with her partner. Nazarpour et al. [22], reported in a study that 61% of postmenopausal women had sexual dysfunction. More than 70% of postmenopausal women in a study by Safaei et al. [23], Had unsatisfactory sexual function.

Considering that the women spend a third of their lives in post-menopause [24]. So, efforts should be made to improve the quality of marital life of postmenopausal women. Self-care education is one of the ways that can be employed on the improvement of women’s knowledge and attitude towards menopause. This method of education is defined as the activities of individuals, families and communities with the aim of promoting health, preventing or limiting disease, and restoring health [25]. Although many studies have been done on postmenopausal women and their quality of life and even quality of marital relationship, but these studies have been more descriptive and have not paid attention to the issue of self-care education [26–29]. Therefore, we conducted this randomized, controlled clinical trial study to evaluate the effect of self-care education program on the severity of menopause symptoms and marital satisfaction in postmenopausal women.

## Materials and methods

### Study design

This prospective, randomized, controlled clinical trial study was conducted at gynecology clinic of Baqiyatallah and 502 Artesh hospitals in Tehran, Iran, between 2020 and 2021, to evaluate the effect of self-care education program on the severity of menopause symptoms and marital satisfaction in postmenopausal women. All parts of the study were reviewed according to the Consolidated Standards of Reporting Trials (CONSORT) statement [30]. The protocol study was reviewed and approved by the Ethics Committees of Baqiyatallah University of Medical Sciences (IR.BMSU.BAQ.REC.1399.042). Also, this trial registered in Iranian registry of clinical trials (IRCT20200624047910N1). Written informed consent was obtained from each woman. The study was conducted in accordance with the Declaration of Helsinki and subsequent [31].

### Participants

The study population included postmenopausal women between the ages of 45 and 65 who met all the inclusion criteria. Inclusion criteria include the following; (a) married, (b) at least 12 months have passed since the last menstrual period, (c) no history of physical and mental problems (such as chronic back pain that may be related to osteoporosis, chronic depression that may be related to psychological problems, to do this, we had a panel of specialists, including a psychologist, psychiatrist, obstetrician and gynecologist), (d) normal menopause (not with hysterectomy or oophorectomy), (e) non-smoking and (f) non-participation in research projects in the last three months. Patients were excluded from the study with if not willing to continue participating in the study, absence from more than one training session, hospitalization and change of residence to other cities.

### Sample size

The sample size was calculated according to the comparing the mean of the quality of life before (19.30 ± 6.22) and after (24.1 ± 8.60) an education intervention among postmenopausal women in Iran [32]. Type I error (α) set as two-sided 5% (Z_1−α/2_ = 1.96), type II error (β) set as 20% (Z_1−β_ = 0.84) and power of 80%. The final sample size for each group was considered 26 subjects. According to the nature of the clinical trial study and the probability of reducing the sample size, a 10% drop was considered as attrition rate. So, in each group we considered 35 people.

$$n = \frac{{\left( {\sigma 1^{2} + \sigma 2^{2} } \right) + \left( {{\text{Z}}1 - \frac{{\upalpha }}{2} + {\text{Z}}1 - \beta } \right)^{2} }}{{\left( {\mu 1 - \mu 2} \right) 2}}$$

µ1: mean of the quality of life before intervention (19.30), µ2: mean of the quality of life before intervention (24.1), σ1: SD of the outcome before intervention (6.22).

### Sampling method and randomization

In the present study, the convenience sampling method has been used. Postmenopausal married women between the ages of 45 and 65 who met all inclusion criteria were randomly divided into two groups (intervention or control) based on random block. Block randomization was performed using sealed envelope technique and computer-generated random numbers by Random Allocation Software © (RAS; Informer Technologies, Inc., Madrid, Spain).

### Data collection

Characteristics of the participants were recorded and evaluated based on a checklist of socio-demographic characteristics including age, age of first menstruation, age of menopause, number of children, occupation and educational level. In addition, to assess the severity of menopausal symptoms as well as marital satisfaction as outcomes in pre- and post-intervention, we used Menopause Symptoms' Severity Inventory (MSSI-38) questionnaire [33], and the Revised Dyadic Adjustment Scale (RDAS) designed by Busby et al. [34], which assesses seven dimensions of couple relationships, respectively.

### Intervention procedure

The intervention group received self-care education in physical, psychological, social and sexual dimensions in 5 face-to-face sessions of 45–120 min in groups of 3–5 individuals during a week. While the control group had 5 face-to-face sessions of 45–120 min, they were exactly the same as the intervention group, except that they received only routine care and training about the Coronavirus disease 2019 (COVID-19). In the control group, the content of education was determined due to the COVID-19 pandemic crisis and the need for training in this field, which included improving knowledge about the COVID-19 pandemic and increasing their capabilities in effective management of this crisis. Tutorials include videos, booklets, PowerPoint slides and brochures and pamphlets were about COVID-19 and its preventive measures, which were approved by infectious disease specialists. These items include causes of COVID-19, symptoms, transmission and prevention, instructions, vaccination. Control group educational content sessions are available in Additional file 1: Table S1.

The self-care program for intervention group includes exercise and physical activity, nutrition and diet, relaxation during menopause, and awareness of marital relationships and how to communicate with your spouse. The first session discussed in detail the introduction of the female reproductive system and the definition of menopause and self-care during menopause and its importance. In the second session, the necessary training was given on self-care in osteoporosis, hot flashes and night sweats. In the third session, while reviewing the previous topics and answering the questions of the previous sessions, the participants were given the necessary training about self-care about marital relationships during menopause, self-care about genitourinary problems. In the fourth session, self-care in osteoporosis, self-care in cardiovascular disease, self-care about nutrition and self-care about exercise and physical activity were given. In the fifth session, while reviewing the previous issues, self-care in fatigue and sleep problems, psychological self-care and self-care in mood changes (Interventional group educational content sessions are available in Additional file 1: Table S2). The educational content was prepared by referring to the available library resources and with the guidance of supervisors and consultants. The validity of the educational content was evaluated qualitatively by a survey of 3 faculty members related to the subject under study, including four reproductive health specialists, one sex therapist, two gynecologists and two psychologists. The approved educational content was designed as a booklet and provided to individuals at the end of the training sessions. Also, the educational content presented in each session was prepared in the form of a pamphlet and was given to the participants for review at the end of each training session. In addition, the intervention group provided access to the researcher to ask questions via mobile phone, email and WhatsApp group. At the end of the training period and one month later, the relevant questionnaires were completed by the intervention and control groups.

### Outcome measurements

Menopause Symptoms' Severity Inventory (MSSI-38) questionnaire [33], was used for assessment menopause symptoms in 38 items, organized in 12 sets (depressive mood; anxiety; cognitive impairment; aches and pain; skin and facial hair changes; numbness; body shape changes; perceived loss of control; mouth, nails and hair changes; vasomotor, urinary, and sexual symptoms). Both the frequency and intensity of the symptoms are measured in reference to the previous month, using a 5-point Likert-type scale (from 0 to 4), which ranges from “never” to “daily or almost every day” and from “not intense” to “extreme intensity” respectively. The severity of each symptom is calculated using the mean between the values of frequency and intensity for each of the symptoms. The validity and reliability of MSSI-38 questionnaire was assessed by Hoseinzadeh et al. [35], with Cronbach’s alpha, 0.77. The reliability of the MSSI-38 questionnaire was assessed in this study by test–retest and Cronbach’s alpha, 0.84 and ICC: 0.79 respectively.

Revised Dyadic Adjustment Scale (RDAS) by Busby et al. [34], to assess the quality of marital life, was used in the study. RDAS) is a self-report questionnaire that assesses seven dimensions of couple relationships with 14 items and 3 subscales of categories including agreement (6 questions), satisfaction (5 questions) and cohesion (3 question). Scores on the RDAS range from 0 to 69 with higher scores indicating greater relationship satisfaction and lower scores indicating greater relationship distress. The cut-off score for the RDAS is 48 such that scores of 48 and above indicate non-distress and scores of 47 and below indicate marital/relationship distress. The reliability of the Marital Quality Scale of Busby et al.'s Cronbach's alpha in the Holist, Cody and Miller study for the three subscales of agreement, satisfaction, and cohesion was reported to be 0.79, 0.80, and 0.90, respectively. The reliability of the RDAS questionnaire was assessed in this study by test–retest and Cronbach’s alpha, 0.85.

### Statistical analysis

The normality of the entered data was checked and confirmed by Kolmogorov–Smirnov test and measures of skewness and kurtosis. To describe and summarize the study results, mean ± standard deviation (SD) for continuous variables and frequency (percentages) for categorical variables were used. A chi-square or Fisher’s exact test was performed for comparisons between two groups of categorical data. However, continuous data were statistically analyzed using a t-test. Mean score of MSSI-38 questionnaire for severity of menopause symptoms and RDAS for marital satisfaction were computed at the pre- post-intervention for two groups of study. The between group comparisons of baseline measures and demographic variables was done by independent t-test and/or Chi-square test where appropriate. For within group comparisons paired t-test was used for the Numeric Normal variables, respectively, where before and after intervention measurements were taken. For simultaneous assessing the group and time effect and their interaction, two-way analysis of variance with repeated measures (RMANOVA) were used, followed by Sidak post hoc tests. The assumption of sphericity was addressed by Mauchly's test of sphericity and when the assumption was not satisfied the Greenhouse-Geiser correction of P-value were utilized. To assess the effect of intervention, the analysis of covariance (ANCOVA) was used controlling for baseline measures and confounders in a two-step hierarchical model. The correlation between severity of menopause symptoms and marital satisfaction was assessed using a simple linear regression and Pearson correlation coefficient test. In addition, univariate and multivariate logistic regression was employed to estimate the odds ratio (OR) to investigate the independence of risk factors for severity of menopause symptoms and marital satisfaction. All data were analyzed using the Statistical Package for the Social Sciences (SPSS) 21.0 statistical package (Chicago, IL, USA) and two-side *P* < 0.05 indicated a statistically significant difference.

## Results

### Participants of study

**Fig. 1:**
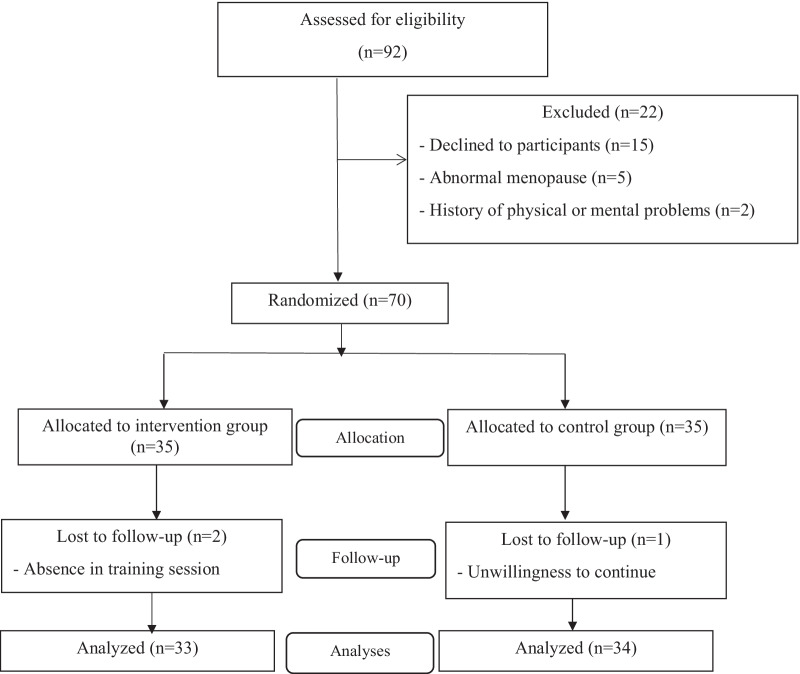
The study flow chart

A total of 70 postmenopausal married women between the ages of 45 and 65 participated in this study. The enrollment flow chart of patients is presented in Fig. 1. Ninety-two postmenopausal women, who referred to the gynecology clinic of Baqiyatallah and 502 Artesh hospitals in Tehran, Iran, screened for eligibility criteria. Out of 92 cases, 22 subjects were excluded from the study due to the not willing to continue participating in the study (n = 15), abnormal menopause due to the hysterectomy or oophorectomy (n = 5) and history of physical or mental problems (n = 2). The remaining seventy eligible patients were randomly allocated into two equal groups (n = 35); Intervention group (received self-care education) and control group (received only routine care and training). During the intervention and follow-up stages, one participant from the control group was excluded due to unwillingness to continue cooperation and two people in the intervention group were excluded due to not attending more than one training session. Finally, 67 postmenopausal married women; 33 in the intervention group and 34 patients remained in the control group for analysis.

### Socio-demographic characteristics

Table 1 shows the socio-demographic characteristics of the study participants in two groups of study. The mean ± SD age, age of first menstruation and age of menopause were 54.50 ± 4.43, 11.50 ± 1.59 and 49.70 ± 3.20 years in participants of study, respectively. Regarding the educational status of the respondents, 41 (61.2%) of the respondents had a diploma. There were no statistically significant differences in baseline social-demographic characteristics of patients between two groups of study such as; age (*P* = 0.256), age of first menstruation (*P* = 0.183), Age of menopause (*P* = 0.068), number of children (*P* = 0.230), occupation (*P* = 0.914), and level of education (*P* = 0.361).

**Table 1:** Socio-demographic characteristics in two groups of study

Variables	Total participants (n = 70)	Intervention group (n = 33)	Control group	*P*-value
Age				
Mean ± SD, (years)	54.50 ± 4.43	53.87 ± 4.50	55.11 ± 4.34	0.256*
Range, (years)	(48–64)	(48–62)	(48–64)	
Age of first menstruation				
Mean ± SD, (years)	11.50 ± 1.59	11.24 ± 1.78	11.76 ± 1.37	0.183*
Range, (years)	(9–15)	(9–15)	(9–14)	
Age of menopause				
Mean ± SD, (years)	49.70 ± 3.20	50.42 ± 3.09	49.00 ± 3.19	0.068*
Range, (years)	(42–58)	(46–58)	(42–58)	
Number of children				
One (%)	18 (26.9)	7 (21.2)	11 (32.4)	0.230**
Two (%)	17 (25.4)	8 (24.2)	9 (26.5)	
Three (%)	25 (37.3)	12 (36.4)	13 (38.2)	
More than three (%)	7 (10.4)	6 (18.2)	1 (2.9)	
Occupation				
Housewife (%)	44 (65.7)	21 (63.6)	23 (67.6)	0.914**
Employee (%)	14 (20.9)	8 (24.2)	6 (17.6)	
Retired (%)	4 (6)	2 (6.1)	2 (5.9)	
Self-employment (%)	5 (7.5)	2 (6.1)	3 (8.8)	
Educational level				
Under diploma (%)	19 (28.4)	7 (21.2)	12 (35.3)	0.361**
Diploma (%)	41 (61.2)	23 (69.7)	18 (52.9)	
University education (%)	7 (10.4)	3 (9.1)	4 (11.8)	

*Independent sample t-test (equal variances assumed base on Levene's Test for Equality of Variances (p > 0.05)

**Fisher's Exact test

### Effect of self-care education on the severity of menopause symptoms and marital satisfaction

**Fig. 2:**
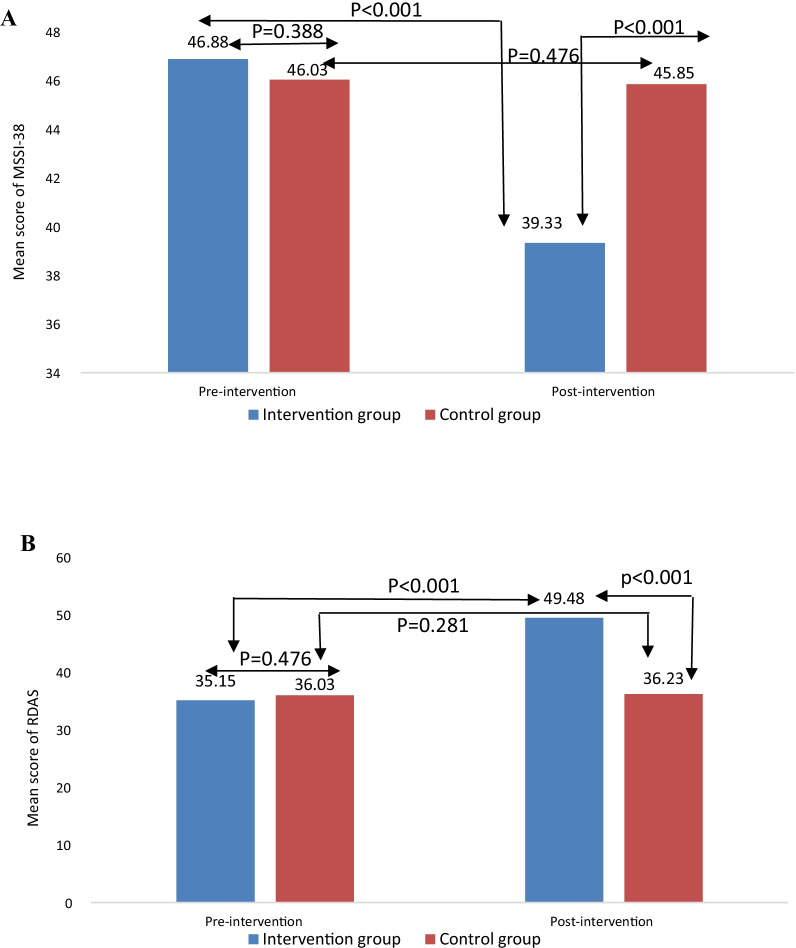
**A** Severity of menopausal symptoms and **B** marital satisfaction in the intervention and control groups pre- and post-intervention

Based on the results of independent t-tests at pre-intervention, there were no significant differences between the two groups in terms of severity of menopause symptoms (46.88 ± 3.3 vs. 46.03 ± 4.6, *P* = 0.388) and marital satisfaction (35.15 ± 4.3 vs. 36.03 ± 5.59, *P* = 0476), which indicates the homogeneity of participants in the study (Table 2). However, post-intervention results based on t-test between two groups showed significant differences in both of severity menopause symptoms (39.33 ± 3.7 vs. 45.85 ± 4.3, *P* < 0.001) and marital satisfaction (49.48 ± 3.2 vs. 36.23 ± 5.69, *P* < 0.001) parameters. According to within group comparison using paired t-test, there was a significant difference in the severity of menopausal symptoms (46.88 ± 3.3 vs. 39.33 ± 3, *P* < 0.001) and marital satisfaction (35.15 ± 4.3 vs. 49.48 ± 3.2, *P* < 0.001) before and after the intervention in the intervention group (Table 2). While, no significant difference was observed in the control group before and after the intervention in terms of severity menopause symptoms (46.03 ± 4.6 vs. 45.85 ± 4.3, *P* = 0.475) and marital satisfaction (36.03 ± 5.59vs. 36.23 ± 5.69, *P* = 0.281). All details are shown in Fig. 2.

**Table 2:** Severity of menopausal symptoms and marital satisfaction in the intervention and control groups before and after the intervention

Variables	Time	Groups		Mean difference (95% CI)	*P*-value	*P*-value (group × time interaction effect)
		Intervention group (n = 33)	Control group (n = 34)			
Severity of menopausal symptoms	Pre-Intervention $	46.88 ± 3.3	46.03 ± 4.6	0.849 (− 1.10 to 2.80)	0.388	< 0.001##
	Post-intervention $$	39.33 ± 3.7	45.85 ± 4.3	7.191 (5.95–8.42)	< 0.001	< 0.001###
	Difference between pre- and post-intervention $$$	7.54 (6.32–8.77) *P* < 0.001	0.176 (− 0.32 to 0.67) *P* = 0.475	7.43 (5.99–8.88)	< 0.001#	
Marital satisfaction	Pre-Intervention $	35.15 ± 4.3	36.03 ± 5.59	− 0.88 (− 3.32 to 1.57)	0.476	< 0.001##
	Post-intervention $$	49.48 ± 3.2	36.23 ± 5.69	13.94 (12.7–15.14)	< 0.001	< 0.001###
	Difference between pre- and post-intervention $$$	14.3 (13.05–15.61) *P* < 0.001	0.206 (− 0.17 to 0.58) *P* = 0.281	14.09 (12.7–15.45)	< 0.001#	

$: Pre intervention based on independent t-test; $$: Post intervention based on Analysis of covariance (ANCOVA) adjusted for pre intervention; $$$: Changes in pre and post intervention in intervention and control groups based on Sidak post hoc in RMANOVA after Greenhouse-Geiser correction; #: Post-intervention based on Analysis of covariance (ANCOVA) adjusted for, Age, First menstruation age, Menopausal age, Number of children, occupation, and education level (fully adjusted model); ##: Assessing the interaction effect of group and time based on two way analysis of variance with repeated measures (RMANOVA) after Greenhouse-Geiser correction; ###: Assessing the interaction effect of group and time based on two way analysis of covariance with repeated measures (RMANCOVA) after Greenhouse-Geiser correction for Age, First menstruation age, Menopausal age, Number of children, Marriage status, Job status, and qualification (fully adjusted model)

The results of ANCOVA for post-intervention measures adjusted for, age, age of first menstruation, age of menopause, number of children, occupation and educational level as the confounders showed statistical difference mean score of MSSI-38 and RDAS questionnaires between two groups. According to the results of two ways ANOVA with repeated measure, there was a significant time effect on severity menopause symptoms (*P* < 0.001) and marital satisfaction (*P* < 0.001). Additionally, comparisons between groups with respect to the interaction of time and intervention showed significant differences for severity menopause symptoms (*P* < 0.001) and marital satisfaction (*P* < 0.001).

### Correlation analysis

Pearson correlation coefficient test showed a significant inverse correlation between severity of menopausal symptoms and marital satisfaction after intervention with r = -0.461, *P* < 0.001. Moreover, a simple linear regression was carried out to test if changes in severity of menopause symptoms significantly predicted the status of marital satisfaction. The results of the regression indicated that the model explained 21.2% of the variance and that the model was significant, F (1, 65) = 17.501, *P* < 0.001. It was found that the severity of menopause symptoms significantly predicted marital satisfaction (β_1_ = -0.294, *P* < 0.001). The final predictive model was: Proportion of increased marital satisfaction = 55.21 + (-0.294* severity of menopausal symptoms).

### Univariate and multivariate logistic regression analysis

Univariate and multivariate logistic regression analysis to determine the independent factors associated with severity of menopausal symptoms and marital satisfaction are presented in Table 3. The score of severity of menopausal symptoms based on MSSI-38 scale as dependent variable was divided into two categories ≤ 43 as low and ≥ 44 as high. Furthermore, the score of marital satisfaction based on RDAS scale as dependent variable was divided into two categories ≤ 47 as marital/relationship distress and ≥ 48 as non-distress. Based on multivariate logistic regression analysis, pre-intervention score of severity of menopausal symptoms was independently associated with marital satisfaction (OR: 1.483, 95% CI: 1.167–1.886, *P* < 0.001).

**Table 3:** Univariate and multivariate logistic regression analysis to determine the independent factors associated with severity of menopausal symptoms and marital satisfaction

Dependent variables	Independent variables	Univariate		Multivariate	
		OR (95% CI)	*P*-value	OR (95% CI)	*P*-value
Severity of menopausal symptoms	Age	1.094 (0.976–1.226)	0.123	1.112 (0.930–1.329)	0.246
	Age of first menstruation	0.995 (0.733–1.351)	0.974	0.815 (0.505–1.315)	0.402
	Age of menopause	0.882 (0.749–1.039)	0.133	0.886 (0.687–1.141)	0.348
	Number of children	0.837 (0.508–1.378)	0.484	1.204 (0.529–2.741)	0.658
	Educational level (higher diploma vs. diploma)	1.200 (0.209–6.884)	0.838	1.053 (0.073–15.15)	0.969
	Educational level (diploma vs. under diploma)	0.853 (0.168–4.326)	0.848	1.239 (0.112–13.69)	0.861
	Pre-marital satisfaction	1.138 (1.018–1.274)	0.024*	1.395 (1.150–1.692)	0.001*
Marital satisfaction	Age	0.955 (0.852–1.071)	0.434	0.892 (0.743–1.07)	0.219
	Age of first menstruation	0.865 (0.631–1.186)	0.368	0.840 (0.583–1.209)	0.348
	Age of menopause	1.097 (0.935–1.287)	0.255	1.130 (0.892–1.432)	0.311
	Number of children	1.323 (0.791–2.211)	0.286	1.542 (0.802–2.966)	0.194
	Educational level (higher diploma vs. diploma)	5.182 (0.572–46.96)	0.144	5.036 (0.448–56.66)	0.191
	Educational level (diploma vs. under diploma)	2.143 (0.204–22.478)	0.525	1.579 (0.114–21.81)	0.733
	Pre-severity of menopausal symptoms	1.109 (0.972–1.274)	0.124	1.483 (1.167–1.886)	0.001*

The score of Severity of menopausal symptoms based on MSSI-38 scale as dependent variable was divided into two categories ≤ 43 as low severity and ≥ 44 as high severity; The score of marital satisfaction based on RDAS scale as dependent variable was divided into two categories ≤ 47 as marital/relationship distress and ≥ 48 as non-distress

*P < 0.05 considered as significantly

## Discussion

This study aimed at investigating the effect of self-care education program on the severity of menopause symptoms and marital satisfaction in postmenopausal women. The study results demonstrated the positive impact of self-study educational intervention on reduce the severe menopause symptoms and increase the marital satisfaction. In the current study, the mean scores related to the severity of menopausal symptoms and marital satisfaction improved after the intervention of self-care education. Learning self-care activities can lead to health and well-being, increase adaptation to symptoms, and increase marital satisfaction. Acquiring Knowledge about menopause parameters is an initial phase for the development of positive behavior and improvement of menopausal women’s quality of life [36–39]. When women’s awareness regarding menopause increases, it improves their attitude, health behavior, health habits and health care which eventually lead to an improvement in their quality of life as well as marital satisfaction [40].

One aspect of quality of life is the quality of marital relationships that may be disrupted during menopause [17, 18]. In this regard, Abedzadeh et al. [41], they pointed out that women's quality of life during menopause is inadequate and marital satisfaction is a factor related to quality of life. Also, the results of study by Ramezani et al. [42], showed that reducing the satisfaction of marital relations during menopause can reduce the quality of life of women and the overall satisfaction of postmenopausal women in married life. In the present study, self-care education based on exercise and physical activity, nutrition and diet, relaxation during menopause with a focus on the educational needs of marital relationships led to awareness in patients. The study of Ruggirero et al. [43], described the effect of self-care education on monitoring nutrition and physical activity to control body mass index (BMI) during menopause period. Similarly, Kong et al. [44], showed the effect of combined dietary regimen and physical activity as self-control behaviors for weight loss in postmenopausal women. In addition, Strudee et al. [45], reported that lifestyle modifications, especially exercise and physical activity, can be effective in reducing hot flashes in postmenopausal women, which is their main complaint, and improving their quality of life.

On the other hand, sexual dysfunction in postmenopausal women is one of the factors that affect marital satisfaction. Mirmohammad Aliei et al. [46], suggest that four sequential weekly educational sessions can improve sexual dysfunction in menopausal women suffering from sexual dysfunction. The advantage of our study with this study is that our self-care training program was performed in eligible postmenopausal women without considering sexual dysfunction, and this program was able to prevent sexual dysfunction by promoting self-care, and this shows the importance of self-care.

The importance of self-care program lies in the fact that it increases the autonomy of patients and improves the ability of patients to take care of themselves. Taking self-care courses will make menopausal participants accept it as a new stage in their lives and get enough information about self-care specific to this period of lives. Therefore, the present study is consistent with the previous studies in that the training affects the changes in lifestyle, awareness of menopause symptoms and consequently improving marital relationship [3, 43, 47]. Despite the differences in the target groups, methodology, types of dimensions of quality of life, content of interventions and also the method of intervention, but it can be said that the use of educational methods and especially self-care training has been effective in improving menopausal symptoms. One of the limitations of this study was the small sample size, which may not be generalizable to other groups and communities. In general, due to the coincidence of this study with the time of the outbreak of COVID-19, there were few postmenopausal women referred in the clinic, and on the other hand, holding training sessions in accordance with health protocols was a difficult task that we had to hold sessions for 4–5 people. Therefore, we suggest that this study be performed at a more appropriate time in a wider range.

## Conclusions

In summary, the results of study demonstrated the positive impact of self-study educational intervention on reduce the severe menopause symptoms and increase the marital satisfaction. Adequate knowledge about the definition of menopause and its parameters along with training in self-care against various menopausal symptoms is very useful to adapt to this stage of life. The favorable attitude of women towards menopause and full awareness of their physical and mental care during this period, has a significant effect on reducing menopausal symptoms and thus increasing marital satisfaction. Therefore, we recommend that more attention be paid to providing self-care educational content to improve the quality of life in postmenopausal women.

## Supplementary Information

**Additional file 1.** Intervention procedure for experimental and control groups. **Table S1:** Control group educational content sessions. **Table S2:** Intervention group educational content sessions.

## Data Availability

The data that support the findings of this study are available from the corresponding author upon reasonable request.

## Abbreviations

MSSI-38: Menopause Symptoms' Severity Inventory questionnaire
RDAS: Revised Dyadic Adjustment Scale questionnaire
FSH: Follicle-Stimulating Hormone
LH: Luteinizing Hormone
OR: Odds Ratio
ANCOVA: Analysis of Covariance
RMANOVA: Repeated Measures Analysis of Variance
