# Supplementary material for: The effect of self-care education program on the severity of menopause symptoms and marital satisfaction in postmenopausal women: a randomized controlled clinical trial
Source: BMC Womens Health. 2022 Mar 14;22:71. doi: 10.1186/s12905-022-01653-w (PMC8919913; doi:10.1186/s12905-022-01653-w)
Supplement: Supplementary file 1 — Additional file 1. Intervention procedure for experimental and control groups. Table S1: Control group educational content sessions. Table S2: Intervention group educational content sessions. [file 12905_2022_1653_MOESM1_ESM.docx]

**Table 1: Control group educational content sessions**

| **Sessions** | **Educational content of control group meetings** | **Educational method** | **Training material** |
| --- | --- | --- | --- |
| **1** | Information about the main features of COVID-19 | Lecture | PowerPoint slides |
| **2** | Epidemiology and etiology of the necessary information about COVID-19 and effects of COVID-19 on daily life | Lecture | PowerPoint slides and Training video |
| **3** | Preventive measures against COVID-19 disease, guidelines and preventive measures against the COVID-19 disease | Virtual webinar and group discussion | PowerPoint slides and Training video |
| **4** | Strategies and coping principles during stressful conditions brought about by COVID-19 disease | Lecture | PowerPoint slides and  audio files |
| **5** | Necessary information on the vaccination during COVID-19  Provide information on how to improve the quality of life during the Covid-19 crisis | Lecture | Web-based brochures, pamphlets and PowerPoint  slides |

**Table 2: Intervention group educational content sessions**

| **Sessions** | **Main content** | **Recommendation for educational content** | **Training material** | **Educational method** |
| --- | --- | --- | --- | --- |
| **1** | Introduction of female reproductive system and definition of menopause | **(A)** Menopausal self-care and its importance, **(B)** Brief description of the uterus, ovaries, cervix, vagina, fallopian tubes and a brief overview of the function of these organs in simple and understandable language | Lecture | - PowerPoint slides  - Medical modeling  - Training video |
| **2** | Self-care in hot flashes and night sweats | **(A)** limit caffeine, reduce salt intake, fatty foods, sugar and caffeinated foods, **(B)** Wear thin clothing to cool down faster during hot flashes, especially at night, **(C)** No smoking, **(D)** Regular exercise and physical movements; walking, swimming, yoga, and pelvic exercises, **(E)** Consume large amounts of calcium , **(F)** If necessary, use hormone therapy with the opinion of the treating physician | Virtual webinar and group discussion | - PowerPoint slides  - Training video  - Brochures and pamphlets |
| **3** | Self-care about marital relationships during menopause and genitourinary problems | **(A)** Tips for maintaining post-menopausal marital relationships, **(B)** Having regular sex, **(C)** Using vaginal lubricants and moisturizers such as K-Y gels or water-soluble lubricating gel to reduce vaginal dryness will increase libido, **(D)** Kegel exercise, **(E)** Intimacy with your spouse, **(F)** In case of severe decrease in sexual desire and lack of effect of the said care, it is recommended to see a doctor | Lecture | - PowerPoint slides  - Training video |
| **4** | Self-care in osteoporosis, in cardiovascular disease, in nutrition, about exercise and physical activity | Recommended for consumption: **(A)** Calcium-rich foods such as 3-4 glasses of low-fat milk or yogurt or cheese or 600-500 grams of parsley or turnip, carrots, oranges, legumes, cabbage, citrus fruits, tuna, lettuce and dates, **(B)** Reduce the number of meals and food portions, **(C)** Avoid eating fatty and fried foods, **(D)** Recommended consumption of olive oil, **(E)** Vitamin D; 15 minutes of direct sunlight daily, **(F)** Quit smoking and consume less tea, coffee and red meat, sugar, salt and canned foods, **(G)**  Adequate consumption of fluids (8 glasses per day) and high-fiber material, **(H)** Having continuous and balanced physical activity, **(I)** Correction of sitting, standing and bending posture, **(J)** Bone density tests every 3-5 years to diagnose and follow up on osteoporosis treatment | Lecture | - PowerPoint slides  - Audio file |

**Continue Table 2: Intervention group educational content sessions**

| **Sessions** | **Main content** | **Recommendation for educational content** | **Training material** | **Educational method** |
| --- | --- | --- | --- | --- |
| **5** | Self-care in fatigue and sleep problems, psychological and in mood changes | **(A)** In case of lack of sleep, eat a glass of milk before going to bed, **(B)** Perform light sports activities for at least 30 minutes a day, **(C)** Have a healthy diet rich in nutrients, **(D)** Positive attitude towards menopause, **(E)** Be helpful and accept aging changes, **(F)** Get enough sleep, **(G)** Tell others about your experiences and what you have learned, **(H)** Rely on yourself and do not depend on others, **(I)** Be diligent. Have big goals. Forget this sentence that is past us and too late for our age.  **(J)** Try to learn something new at every opportunity “It is never too late to learn”, **(K)** Do not take life hard on yourself and others, **(L)** Avoid nagging and getting too much criticism.  **(M)** Smiling, **(N)** Get help from a consultant for confusion, mental disorders and memory impairment, **(O)** Performing regular and appropriate physical, **(P)** accepted the realities and developments of life and was interested in life and enjoyed it | Virtual webinar and group discussion | - PowerPoint slides  - Brochures and pamphlets |
